# Phytochemicals, Health-Promoting Effects, and Enzyme Inhibition Traits of *Phlomis stewartii* Extracts

**DOI:** 10.3390/molecules29051049

**Published:** 2024-02-28

**Authors:** Mamoon Ur Rasheed, Syed Ali Raza Naqvi, Fahad Al-Asmari, Muhammad Abdul Rahim, Mohamed Fawzy Ramadan

**Affiliations:** 1 Department of Chemistry, Government College University, Faisalabad 38040, Pakistan; mamoonsandhu68@gmail.com; 2 Department of Food and Nutrition Sciences, College of Agricultural and Food Sciences, King Faisal University, Al-Ahsa 31982, Saudi Arabia; falasmari@kfu.edu.sa; 3 Department of Food Science & Nutrition, Faculty of Medicine and Allied Health Sciences, Times Institute, Multan 60700, Pakistan; mabdulrahim@t.edu.pk; 4 Department of Clinical Nutrition, Faculty of Applied Medical Sciences, Umm Al-Qura University, Makkah 24382, Saudi Arabia

**Keywords:** plant extract, phytochemicals, biological activity, antioxidants, phenolic acids, diabetes

## Abstract

*Phlomis stewartii* is a wild, perennial woody plant used for diverse therapeutic targets. The present work evaluated the influence of independent variables such as extraction time, solvent concentration, and speed in the range of (100 mL, 150 mL, and 200 mL), (2 h, 5 h, and 8 h), and (100 rpm, 150 rpm, and 200 rpm), respectively, on extraction yields, phytochemical components, total phenolic contents (TPC), and total flavonoid contents (TFC) of *P. stewartii* extract. In the present work, response surface methodology (RSM) was applied to optimize the extraction yield. High-performance liquid chromatography (HPLC) was performed to detect the bioactive constituents of the extracts. The potent extracts were analyzed to study α-amylase and α-glucosidase inhibitory activities. Under the optimized conditions of solvent concentration (200 mL), extraction time (8 h), and speed (150 rpm), the whole plant methanol extract (WPME) showed a maximum extraction yield of 13.5%, while the leaves methanol extract (LME) showed a maximum TPC of 19.5 ± 44 mg of gallic acid equivalent (GAE) per gram of extract and a maximum TFC of 4.78 ± 0.34 mg of quercetin equivalent (QE) per gram of extract. HPLC analysis showed the presence of p-coumaric, gallic acid, quercetin, salicylic acid, sinapic acid, and vanillic acid. LME showed the highest α-amylase inhibitory activity (IC_50_ = 46.86 ± 0.21 µg/mL) and α-glucosidase inhibitory activity (IC_50_ value of 45.81 ± 0.17 µg/mL). Therefore, in conclusion, LME could be considered to fix the α-amylase and α-glucosidase-mediated disorders in the human body to develop herbal phytomedicine.

## 1. Introduction

Autoxidation and oxidative stress of human lipoproteins and lipids induce toxic compounds that result in human health problems such as aging, neurodegenerative diseases, cardiovascular diseases, diabetes, cancer, and other neural disorders [1]. All these disorders could be countered or even cured by using exogenous compounds. Because of their complexity in human health, the use of these compounds is limited; therefore, attempts have been undertaken to explore natural agents as an alternative to artificial drugs [2]. Phenolics, flavonoids, alkaloids, ascorbic acids, amides, saponins, and various bioactive components from different parts of plants play ample roles in human health because of their biological potential to fix a variety of health disorders. The yield and biological activity of the plant extract are affected by the extraction approach and the nature of the solvent used. Reported research indicates that methanol is highly suitable to extract a high yield of bioactive compounds [3]. Different ultrahigh extraction methods, supercritical carbon dioxide extraction, microwave-assisted extraction, and shaking extraction methods have been used for plant extract preparations [4]. Mechanical shaking extraction is one of the simplest, least costly, and least invasive extraction processes that can provide good yields by optimizing the extraction parameters. RSM is applied to optimize the extraction yield with the least solvent and shaking time possible. Box–Behnken design (BBD) is one type of RSM commonly used to optimize the technical parameters for extraction, and it is frequently used for other approaches required in optimizing a procedure [5].

*Phlomis* is a genus of over 100 species in the family *Lamiaceae*, and *P. stewartii* is one of them that grows in desert areas of Pakistan (Baluchistan) from June to August. The *Lamiaceae* family is known to have strong medicinal compounds that are utilized in herbal remedies for various disorders [6]. However, to the best of our knowledge, the only study that was reported on *P. stewartii* was the isolation of p-hydroxybenzoic acid, notohamosin, caffeic acid, and phenylethanoid and their evaluation as α-glucosidase inhibitors [7]. Therefore, our study aimed to: (a) optimize the parameters for phytochemical extraction from the dried whole plant, leaves, and flower powder using methanol as an extracting solvent; (b) determine the total phenolic and total flavonoid contents; and (c) test the extracts obtained under the best independent variable conditions (LME1, FME1, and WPME1) for in vitro α-amylase and α-glucosidase inhibition activities.

## 2. Results and Discussion

### 2.1. Methanolic Extracts

The methanol extracts of *P. stewartii* leaf, flower, and whole plant (dry powder of leaf, flower, root, and stem) exhibited 8.97%, 10.8%, and 13.5% extraction yields, respectively. The highest yield was observed by WPME, where independent variables 200 mL, 8 h, and 150 rpm were set to study the response. The LME, with independent variables 100 mL methanol, 2 h extraction time, and 150 rpm shaking speed, produced the lowest response to extraction yield i.e. 8.76%. The effect of different sets of independent variables on extraction yield is given in Table 1.

**Figure 1:**
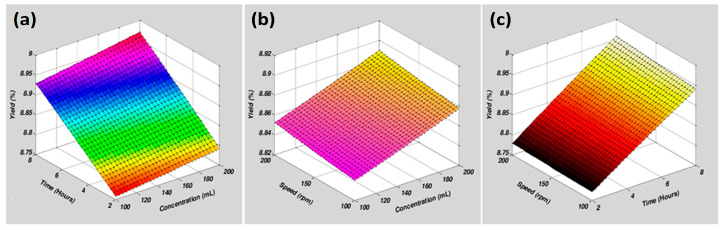
Response surface plots present the mutual interaction’s effect on extraction yield from LM extracts. (**a**) Time vs. concentration; (**b**) Speed vs. concentration; (**c**) Speed vs. time. The green color shows the most optimized area and the red area is the least optimized.

**Figure 2:**
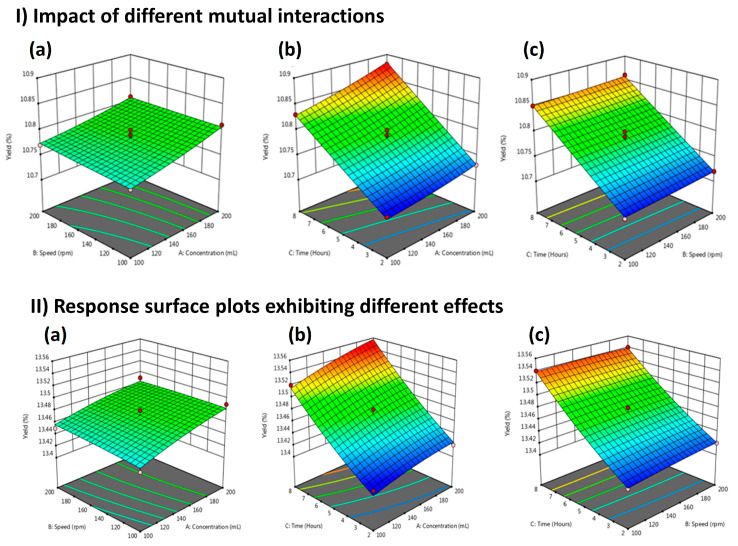
Response surface plots exhibiting; (**I**) the impact of different mutual interactions on FME for extraction yield. (**a**) Time vs. concentration, (**b**) Speed vs. concentration, (**c**) Speed vs. time, and (**II**) response surface plots exhibiting different effects (**a**) Time vs. concentration; (**b**) Speed vs. concentration; and (**c**) Speed vs. time. The green color shows the most optimized area and the red area is the least optimized.

Figure 1 presents the influence of three independent variables on LME. Figure 1a illustrates the response surface plot between extraction time and solvent concentration. Figure 1b shows the mutual relationship between speed and solvent concentration, which results in improved extraction yield. The rate of extraction yield decreased when a mutual interaction was observed between speed and extraction time (Figure 1c). Figure 2 shows the combined effect of independent variables on FME and WPME. Figure 2(I-a) depicts the direct relationship between solvent concentration and speed. Figure 2(I-b) illustrates the mutual interaction between extraction time and solvent concentration. Figure 2(I-c) displays the combined effect of speed and extraction time on extraction yield. The extraction yield of WPME decreased when an interaction was observed between speed and solvent concentration, as shown in Figure 2(II-a). The interaction between extraction time versus solvent concentration and speed versus extraction time is shown in Figure 2(II-b,II-c), respectively.

A previous study reported that other members of the same genus, *P. olivieri* Benth, *P. elliptica* Benth, *P. persica* Boiss, and *P. bruguieri* Dest exhibited extraction yields 7.8%, 5.5%, 8.0%, and 5.9%, respectively which is comparatively lower than the extract of WPME (13.5%) [8]. A recent study reported 8.66% and 9.09% methanol extraction yields from *P. umbrosa* Turcz and *P. megalantha* Diels, respectively, which agrees with our results obtained from LME but is comparatively less than FME and WPME [9]. It has been reported that the methanol extraction yield obtained from *P. Bruguieri*, *P. herba* venti, and *P. Olivieri* was 10.6%, 11.3%, and 9.2%, respectively, which is, in turn, less than our findings [10]. The current study’s findings support previously published information indicating that the genus Phlomis is a Lamiaceae family member and has a strong methanol extraction yield [8].

The beneficial effects of natural products on biological mechanistic control and plant growth have been well documented [11]. Optimized production at the lowest cost is essential for standard disease treatment protocols. RSM is known for the optimization of parameters to reach a set of parameters for maximum production in a statistical way. The current study optimized RSM-based physical parameters such as solvent concentration, extraction time, and speed to reach the maximum extraction yield at the lowest cost. As shown in Figure 1b LME, Figure 2(I-b) FME, and Figure 2(II-b) WPME, yield depends on solvent concentration and time duration. Previously reported study also mentioned that extraction time and solvent concentration are essential in increasing the extraction yield [12]. The polarity of the solvent plays a crucial role in the extraction yield and compounds present in plants [13]. Methanol, in conclusion, has been reported the best solvent for extracting bioactive constituents from plants [3].

### 2.2. Model Fitting

RSM is much better than classic single-factor optimization for medicinal plant extraction. The standard of RSM comprises using fewer experimental measurements, pinpointing interaction amongst variables, and providing a statistical interpretation of verity [14]. Box–Behnken design (BBD) was used to find the interaction among solvent concentration, extraction time, and speed. Table 2 shows the ANOVA for statistical significance of the quadratic regression model equation, independent factors, their interplay, and model fitness.

Moreover, model suitability quality was assessed by probability value (*p*-value), R^2^, adjusted R^2^, predicted R^2^, and lack of fit. The greater the f-value greater than 0.05 for specific independent process variables, the greater the effect of that variable [15]. The satisfaction of the model was checked by the determination of the coefficient (R^2^), whose value lies between 0 and 1, indicating better-predicted values and a stronger model as well [16]. The “Predicted R-Square” and “Adjusted R-Square” calculate the adequacy and quality of the model. Adequate precision indicates the signal-to-noise (S-N) ratio, which should be greater than 4. In this work, the *p*-value of each model is given in Table 2. It could be concluded that three quadratic coefficients (A^2^, B^2^, and C^2^), three linear coefficients (A, B, and C), and three interactive coefficients (AB, AC, and BC) were significant or non-significant, which indicated the scheme of interactions between tested variables. The values of the determination coefficients R^2^ LME, FME, and WPME were 0.9978, 0.9858, and 0.9818, respectively, indicating a reasonable fit of the model to experimental data. The data also shows all the responses of LME yield “Predicted R-Square” values of 0.9909 in a reasonable relationship with the “Adjusted R-square” values of 0.9949, which were less than 0.0046. In FME and WPME yield all responses, “Predicted R-Square” values of 0.9521 and 0.8300 showed agreement with “Adjusted R-Square” values of 0.9676 and 0.9583, which were less than 0.0155 and 0.1283, respectively. For this model, an LME yield of 39.5847, a FME yield of 31.9893, and a WPME yield of 21.7384 indicated an adequate precision signal for the model to be used productively and to be used to navigate the design space. LME, FME, and WPME yield recorded high predicted R-Square values, which supported the highly significant model it was, as reported by a previous study [16]. Regression equations of yield for actual and coded levels using response surface methodology (RSM) for methanol extraction are given in Table 3.

### 2.3. Total Phenolic Contents (TPC)

Among all methanol extracts (LME, FME, and WPME), LME (run 1) exhibited the highest TFC of 19.5 mg GAE/g dry weight (DW) under the influence of extraction conditions such as solvent concentration of 200 mL, extraction time of 8 h, and orbital shaker speed of 150 rpm. At the same time, FME (run 15) recorded the lowest TPC (12.3 mg GAE/g DW) under the influence of independent parameters such as solvent concentration (100 mL), extraction time (2 h), and orbital shaker speed (150 rpm), as given in Table 4.

**Figure 3:**
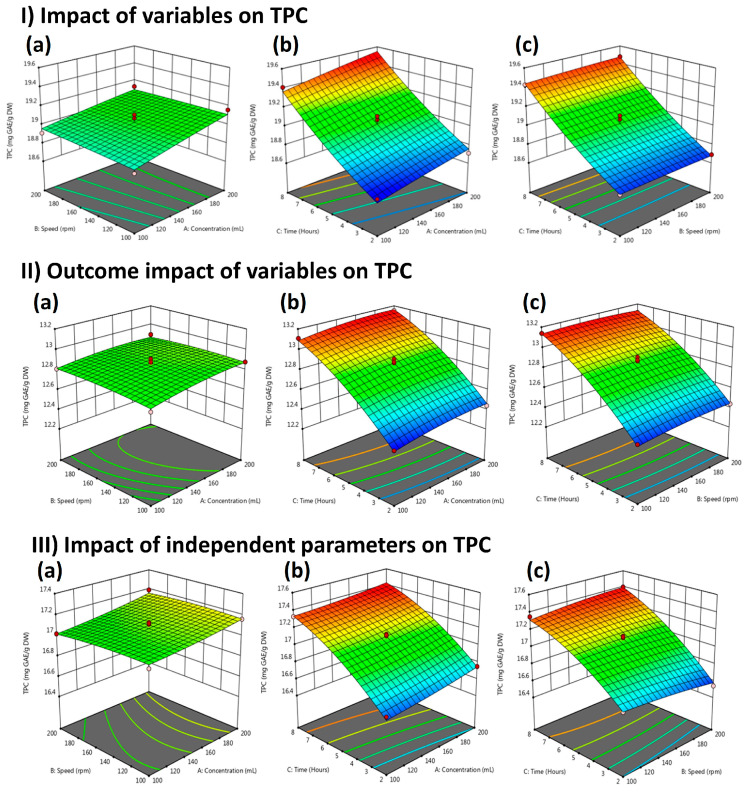
Response surface plots (3-D) showing; (**I**) the impact of variables on TPC present in LME: (**a**) Time vs. concentration; (**b**) Speed vs. concentration; (**c**) Speed vs. time. (**II**) the outcome of variables (speed, solvent concentration, and extraction time) on TPC present in FME: (**a**) Time vs. concentration; (**b**) Speed vs. concentration; (**c**) Speed vs. time; and (**III**) the impact of independent parameters on TPC in WPME: (**a**) Time vs. concentration; (**b**) Speed vs. concentration; (**c**) Speed vs. time. The green color shows the most optimized area and the red area is the least optimized.

Figure 3(I-a–I-c) displays the combined effect of independent variables on LME solvent concentration versus speed, extraction time versus solvent concentration, and extraction time versus speed. The amount of TPC reached its highest value when mutual interaction was found between extraction time and solvent concentration, as shown in Figure 3(I-b). Figure 3(II-a,I-c) shows the response surface plots of FME for the influences of solvent concentration versus speed, extraction time versus solvent concentration, and extraction time versus speed on TPC. As shown in Figure 3(II-b), TPC was increased as a mutual effect was noted between extraction time and solvent concentration, and it started to decrease under the influence of speed and solvent concentration, as shown in Figure 3(II-a). Figure 3(III-a,III-c) illustrates the WPME 3D response surface plot for the influence of extraction time, solvent concentration, and speed on TPC. The result shows that all independent variables have significant effects on TPC. As shown in Figure 3(III-b), the combined effect of extraction time and solvent concentration results in increased TPC compared to the extraction time and speed shown in Figure 3(III-c). Speed and solvent concentration influence decreased the TPC, as shown in Figure 3(III-a).

Compared to other members of this genus, the methanol extract of *P. biloba* yielded TPC (153.46 µg GAE/mg extract) using methanol at room temperature under continuous shaking for 24 h [17]. In another study, *P. samia* methanol extracts contained 73.14 mg GAE/g TPC; this is higher than *P. stewartii* extracts. This could be because the plant is gathered at various times and locations, and the extracted extract contains 80% methanol. [18]. Quantitatively, a previous investigation has mentioned that methanol extracts of *P. umbrosa* and *P. megalantha* recorded good TPC of 39.43 mg GAE/g and 55.20 mg GAE/g, respectively [9]. For comparison purposes, previously investigated plants belonging to the family Lamiaceae contained different TPC levels, such as *P. bruguieri* Desf reported 4.7 mg catechin equivalent per gram dry weight of extracts (mg CE/g DW), *P. persica* Bioss (6.5 mg CE/g DW), *Marrubium vulgare* (4.6 mg CE/g DW), *P. elliptica* Benth (9.0 mg CE/g DW), and *P. olivieri* Benth (9.0 mg CE/g DW) [8]. Higher concentrations of phenolics and flavonoids trigger a particular plant’s biological and pharmaceutical attributes. The current research optimized RSM-based independent variables such as solvent concentration, extraction time, and speed to reach maximum yield at the lowest cost. As shown in Figure 3(I-b) LME, Figure 3(II-b) FME, and Figure 3(III-b) WPME, extraction yield depends significantly on solvent concentration and extraction time, which agrees with previously reported data that extraction time and solvent concentration play a vital role in increasing the extraction yield [12].

The *p*-value of each model is given in Table 5. The determination coefficients R^2^ of LME, FME, and WPME were 0.9762, 0.9932, and 0.9876, respectively, exhibiting a reasonable fit of the model to experimental data. The data also shows all the responses of the LME yield “Predicted R-Square” values of 0.7988 in a rational relationship with the “Adjusted R-square” values of 0.9456, less than 0.1468. Moreover, in the FME and WPME yield all responses, “Predicted R-Square” values of 0.9381 and 0.8726 showed agreement with “Adjusted R-Square” values of 0.9844 and 0.9717, which were less than 0.0463 and 0.0991, respectively. For this model, the LME yield of 18.5059, the FME yield of 31.9893, and the WPME yield of 25.2133 showed an adequate precision signal for the model to be used productively to navigate the design space.

The model regression equations of LME, FME, and WPME, which have both actual and coded levels using response methodology, are shown in Table 6.

### 2.4. Total Flavonoid Contents (TFC) of Methanolic Extracts

The results obtained from the different methanolic extractions of *P. Stewartii* showed that LME extract exhibited the highest contents of flavonoids (4.78 ± 0.34 ^a^ mg QE/g DW). However, a low level of flavonoids (1.85 ± 0.17 ^e^ mg QE/g DW) was indicated by FME, as shown in Table 7. The following order was obtained in comparison between all these ethanolic fractions: LME > WPME > FME.

**Figure 4:**
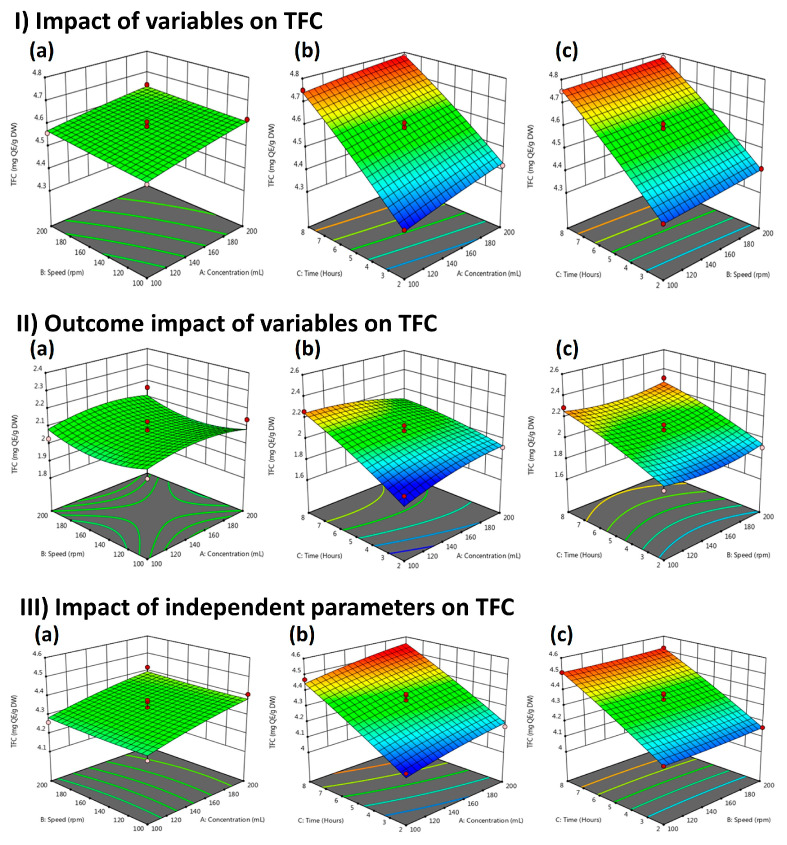
Response surface plots indicating; (**I**) the TFC of LM extracts affected by independent variables in LM extracts: (**a**) Time vs. concentration; (**b**) Speed vs. concentration; (**c**) Speed vs. time; (**II**) the interaction of TFC present in FM extracts: (**a**) Time vs. concentration; (**b**) Speed vs. concentration; (**c**) Speed vs. time; and (**III**) the effects of variables (speed, solvent concentration, and extraction time) on TFC present in WPM extracts: (**a**) Time vs. concentration; (**b**) Speed vs. concentration; (**c**) Speed vs. time. The green color shows the most optimized area and the red area is the least optimized.

The response surface plots of LME extracts between speed, extraction time, and solvent concentration are shown in Figure 4(I-a–I-c). Figure 4(I-a) presents the interaction effects of speed and solvent concentration. Figure 4(I-b) displays that, among all these effects of independent variables, the outcomes of TFC significantly increased under the mutual interaction of extraction time and solvent concentration. Figure 4(I-c) shows the combined effect of extraction time and solvent concentration. Figure 4(II-a) illustrates the combined effect of speed and solvent concentration. Figure 4(II-b) indicated that TFC increased significantly under the influence of extraction time and solvent concentration, while TFC value decreased marginally under the combined impact of extraction time and speed, as shown in Figure 4(II-c). Figure 4(III) shows the combined effect of independent variables on WPM extract. Figure 4(III-a) shows the impact of independent variables (speed and solvent concentration) on TFC. The mutual interaction between the extraction time and solvent concentration is shown in Figure 4(III-b), whereas the response surface plot between extraction time and speed is shown in Figure 4(III-c).

Compared to other members of this genus, the methanol extracts of *P. biloba* leaf and flowers yielded TFC values of 53.8 µg QE/mg and 14.8 µg QE/mg, respectively [17]. Similarly, another study reported that Phlomis plants, such as *P. umbrosa* and *P. megalantha,* revealed the presence of TFC 17.1 epicatechin equivalents per gram (EE/g) of extract and 35.9 g EE/g extract, respectively [9]. Furthermore, previous findings reported that methanol extracts of the *P. samia* plant revealed the presence of 21.61 mg QE/g DW TFC, which is higher than *P. stewartii* extracts [18]. These findings agree with published reports that extraction of the *Phlomis* plant with methanol results in good TFC, which contributes to antioxidant potential [7]. The current research optimized RSM-based physical parameters such as solvent concentration, extraction time, and speed to reach maximum extraction yield at the lowest cost. As shown in Figure 4(I-b) LME, Figure 4(II-b) FME, and Figure 4(III-b), WPME TFC depends on solvent concentration and time duration, which agrees with previously reported data [12].

The *p*-value of each model is given in Table 8. The values of the determination coefficient R^2^ of TFC LME, FME, and WPME were 0.9949, 0.8701, and 0.9647, respectively, exhibiting a reasonable fit of the model to experimental data. The data also shows all the responses of LME yield “Predicted R-Square” values of 0.9600 in a reasonable relationship with the “Adjusted R-square” values of 0.9883, less than 0.0283. Moreover, FME and WPME yielded all responses, and the “Predicted R-Square” value showed agreement with the “Adjusted R-Square” values. For this model, the LME yield of 39.5847, FM yield of 8.9698, and WPME yield of 15.8984 indicated a good precision signal for the model to be used productively and to be used to navigate the design space.

The model regression equations of LME, FME, and WPME, which have both actual and coded levels using response methodology, are shown in Table 9.

### 2.5. HPLC Analysis of Methanolic Extracts

**Figure 5:**
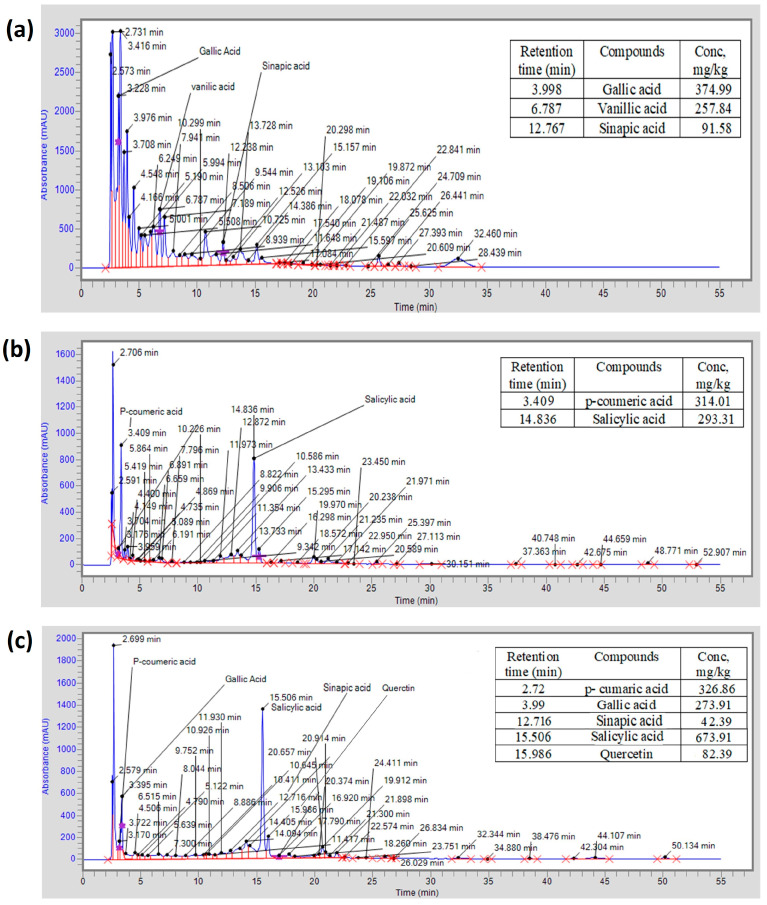
HPLC analysis for detecting phenolic compounds using available standard phenolics and their concentration in *P. stewartii*: (**a**) leaf extract, (**b**) flower extract, and (**c**) whole plant extract. The small and unlabeled peaks are showing the presence of other phytochemicals in the extract that could be detected by using more standard phenolics.

Chromatograms of LME, FME, and WPME for phenolic components are shown in Figure 5. Vanillic acid, gallic acid, and sinapic acid were found in LME; *p*-coumaric and salicylic acid were detected in FME; and salicylic acid, *p*-coumaric, quercetin, gallic acid, and sinapic acid were detected in WPME. HPLC analysis showed results similar to those of the previously reported study using *Phlomis angustissima* and *Phlomis fruticosa* plant methanol extracts [19]. Phenolic compounds inhibit viral proliferation and mediate immunomodulatory and anti-inflammatory activities in the human body [20]. Therefore, the presence of different phenolics in the leaves, flowers, stems, and roots of *P. stewartii* indicates its medicinal value in boosting the immunomodulatory effect and fixing viral diseases. Moreover, sinapic acid has been examined and tested against different pathological conditions such as diabetes, anxiety, oxidative stress, and neurodegeneration [21]. Gallic acid is attributed to its anti-inflammatory, anti-cancer, and anti-inflammatory activities [22]. Vanillic acid has pharmacological traits, including immuno-stimulating, antiapoptotic, hepatoprotective, antioxidant, and neuroprotective properties [23]. It has been reported that quercetin controls the redox balance of the body and increases the expression of catalase (CAT), glutathione (GSH), and superoxide dismutase (SOD) [24]. Various in vitro and in vivo studies have shown that in cells of human and animal models, quercetin showed anti-inflammatory potential.

### 2.6. Enzyme Inhibition

#### 2.6.1. α-Amylase Enzyme Inhibition Activity

The enzyme inhibition activity of LME, FME, and WPME against α-amylase was studied using 25 to 200 µg/mL extract concentrations. At maximum concentration (200 µg/mL), LME showed 83.43% enzyme inhibition, FME showed 81.86%, and WPME showed 81.14%, while acarbose, taken as a control, showed 88.10% α-amylase inhibition activity. The summary of the results is shown in Table 10. LME showed the lowest IC_50_ value (46.86 µg/mL) among all the extracts, followed by FME (58.88 µg/mL) and WPME (53.323 µg/mL), and acarbose showed 33.29 µg/mL. α-Amylase is an important enzyme that hydrolyzes carbohydrates to disaccharides, and α-glucosidase hydrolyzes the disaccharides to monosaccharides like glucose. The inhibition of this enzyme plays a vital role in controlling hyperglycemia and the digestion of carbohydrates to reduce the blood glucose level, which actively leads to diabetes mellitus (DM) [25]. α-Amylase enzyme inhibition activity of LME, FME, and WPME showed medicinal potential to reduce the chance of DM disorder. These results offer a substantial basis for the future use of the *P. stewartii* plant in vivo model in treating and managing DM and the related condition of oxidative stress.

The incidence of DM is rising and becoming a leading health problem with massive economic costs. DM is accompanied by an increased risk factor for morbidity, mortality, respiratory problems, and infertility. The antioxidant defense of DM is lower than that of its normal-weight counterparts, which is not directly associated with central adiposity [26]. Low-grade chronic inflammation is caused by inflammatory aspects such as monocyte chemotactic protein-1, factor-α, and interleukin-6, another major component in the pathogenesis of DM, which may behave synergistically with OS and ROS to induce DM [27].

#### 2.6.2. α-Glucosidase Inhibition Activity

The α-glucosidase inhibitory activity of extracts was also observed using 25 to 200 µg/mL extract concentrations. All three extracts showed dose-dependent α-glucosidase inhibition activity. At 200 µg/mL concentration, the results showed 82.49, 80.22, 81.89, and 87.18% α-glucosidase inhibition by LME, FME, WPME, and acarbose (control). The details of the results at different concentrations are shown in Table 10. Moreover, LME had the lowest IC_50_ value (46.65 µg/mL), followed by FME (56.21 µg/mL), WPME (51.08 µg/mL), and acarbose (37.29 µg/mL). Our results show a similarity in operating α-amylase and α-glucosidase inhibitory action with previously reported studies in which methanol extracts of the *Phlomis* plant show potent α-glucosidase activity and significantly improve the fasting blood glucose level and insulin in diabetic patients. These findings can assist in managing DM disorder [28]. DM is a group of metabolic disorders characterized by abnormal postprandial growth in blood levels. Postprandial hyperglycemia control is considered to be a major issue in the management and treatment of DM. α-Glucosidase secretion from the intestinal chorionic epithelium is responsible for carbohydrate degradation. The α-amylase and α-glucosidase inhibitors slow down the absorption and breakdown of carbohydrates. Consequently, the postprandial blood glucose peak is reduced, and the sugar level is controlled [29].

## 3. Materials and Methods

### 3.1. Collection, Identification, and Preparation of Plant Parts

The *P. stewartii* fresh plant was collected from the desert area of Baluchistan from June to August 2017. The Department of Botany, Government College University, Faisalabad, Pakistan, authenticated the plant. Plant parts (roots, leaves, fruit, and stem) were washed with distilled water and dried in the shade at room temperature for two weeks. Different dried parts were powered by a mechanical blender to be converted into fine powder. Air-tight containers were used to store powder, which was stored in the refrigerator for further use.

### 3.2. Preparation of Plant Extract

For the extraction process, 10 g of dry powder was mixed with different volumes of methanol (100 mL, 150 mL, and 200 mL), and shake at different speeds (100 rpm, 150 rpm, and 200 rpm) for different times (2 h, 5 h, and 8 h) using a shaker. Whatman No. 1 filter papers were used to filtrate the extract mixture. Using a rotary evaporator, the methanol solvent was evaporated in a vacuum at 32 °C. The semisolid matrix was collected, weighed and calculated the extraction yield.

Percentage (%) of extraction yield = Weight of extract after evaporating solvent and freeze drying/weight of dry sample × 100(1)

### 3.3. Total Phenolic Contents (TPC)

TPC was determined using a modified Folin-Ciocalteu method [30]. Briefly, 1 mL of plant extract was mixed with 1 mL of Folin-Ciocalteu reagent and allowed to react at room temperature for 5 min, then 5 mL of Na_2_CO_3_ (1 M) was added. The addition of distilled water to the mixture was adjusted to 10 mL. The solution was incubated at room temperature for 90 min. A spectrophotometer was used to record the absorbance at 760 nm. The standard calibration (0.5, 1, 2, 4, 8 µg/mg) curve was performed using gallic acid. The results were expressed as gallic acid equivalent mg GAE/g dry weight (DW) of extracts.

### 3.4. Total Flavonoid Content (TFC)

TFC was determined using the aluminum chloride method with modifications [29]. In a test tube 0.75 mL of distilled water was added and mixed with a 0.25 mL sample (0.25, 0.5, 1, 2, 4 µg/mg). After this, 0.15 mL of a 5% NaNO_3_ solution was added to the mixture and reacted for 5 min, then 0.3 mL of AlCl_3_ (10%) was added. One milliliter of NaOH was added after 5 min, and the whole mixture was shaken gently. The absorbance of the mixture was recorded at 510 nm in triplicate. Quercetin was standard, and results were given in mg QE/g DW of extract.

### 3.5. HPLC Analysis of Phenolic Acids

For sample preparation for phenolic acid estimation in *P. stewartii* leaves through HPLC, some modifications followed a method reported by [31]. Dried powdered leaf extract (0.5 g) was collected in a flask with a lid along with a 0.5 mL mixture of standard phenolic contents, and then the process of extraction was followed by a 50 mL aqueous mixture of methanol (50% *v*/*v*) for 30 min in an ultrasonic bath. The mixture was centrifuged at 4 °C for 5 min at 3000 rpm. The supernatant was filtrated with a 0.46 µm membrane filter, and a micro syringe injected 20 μL into the HPLC system.

The Perkin Elmer Series 200 HPLC system (Rodgau, Germany) equipped with C-18 column (4.7 × 250 mm, 5 µm stationary phase particle size) and UV/Visible detector was used to analyze the phenolics. For gradient elution, a binary solvent mobile-phase system was selected. The mobile phases of water and methanol were named A and B, respectively. Both phases were acidified by using 0.02% trifluoroacetic acid (TFA). The gradient elution was carried out as follows: 0–4 min, 25% B; 3–8 min, 25–30% B; 7–12 min, 30–50% B; 12–15 min, 50% B; 15–18 min, 50–80% B; 18–22 min, 80% B; 22–25 min, 80–25% B. The 1.0 mL/min flow rate was adjusted at 25 °C column temperature. The detection wavelength of 254 nm was selected.

### 3.6. α-Amylase Inhibition Assessment

The α-amylase inhibition assay of the extract was performed [32]. A 96-well plate combined 50 µL plant extract with 150 µL of (C_6_H_10_O_5_)_n_ solution and 10 µL of the enzyme. The mixture was incubated for 30 min at 37 °C. All plates were closed, and 20 µL of NaOH and 20 µL of color reagent were added; all plates were kept in the water bath at 100 °C for 20 min. The α-amylase study was performed by measuring the mixture’s absorbance at 540 nm with the help of an Elisa plate reader. To adjust the mixture’s absorbance, blank samples were employed in which the enzyme was changed with a buffer solution. A negative control was run, in which extracts were replaced with 50 µL of DMSO, and the maximum potential of the enzyme was checked. Enzyme activity was evaluated using a negative control reaction in which plant extracts were replaced with 50 µL of DMSO. All the interferences from the plant extracts were utilized, including color and C_25_H_43_NO_18_ solution at different concentrations (25, 50, 100, and 200 µg/mL). The following equation calculates the percentage of α-amylase inhibition.

Iα − Amylase = 100 × (A control − A sample)/(A control).(2)

### 3.7. α-Glucosidase Inhibition Assay

The α-glucosidase activity was performed [25]. In a 96-well plate, the reaction mixture having 10 µL α-glucosidase (1 U/mL), 50 µL phosphate buffer (100 mM, pH = 6.8), and 20 µL of different concentrations of the sample (25, 50, 100, 200 µg/mL) was pre-incubated for 15 min at 37 °C. Subsequently, 5 mM of P-NPG (20 µL) was added and incubated for an additional 20 min at 37 °C. Then, 0.1 M of Na_2_CO_3_ (50 UL) was used to stop the reaction. The absorbance of the released C_6_H_5_NO_3_ was noted at 405 nm using a microplate reader. Acarbose was used as a positive control. The sample was set up in parallel as a control. The activity of α-glucosidase plant extract can be defined in % inhibition, obtained using the formula.

Inhibition potential (%) = (1 − As/Ac) 100(3)

where,

As = Absorbance in the presence of a test sample

Ac = Absorbance of control

### 3.8. Optimization Design

For the statistical examination of BBD, 17 runs in experimental conditions, including 5 center points, were preferred for various combinations. The model’s suitability to anticipate the optimum response value for *P. stewartii* leaf extraction has been evaluated using the supreme conditions chosen. The extraction conditions, such as solvent concentration (100 mL, 150 mL, and 200 mL), extraction time (2 h, 5 h, and 8 h), and speed (100 rpm, 150 rpm, and 200 rpm), were optimized. The coded and actual levels of extraction conditions are given in Table 11. The extract was optimized using the shaker and rotary evaporator. Extracts were collected from rotary evaporators for further analysis, such as TPC and TFC.

### 3.9. Statistical Analysis

BBD was selected to find the interactions among solvent concentration, extraction time, and speed. Extraction was statistically examined for its significant value by applying a software package (MATLAB, version 7.5.0.338; R2007a, Natick, MA, USA) as given by [33]. The optimization of 17 runs was carried out in triplicate, and average mean values were disclosed with a standard deviation. Further, one-way ANOVA of statistical analysis with post hoc was applied for in vivo studies. The data were shown as mean ± SE with a 5% significance level and superscripts of different alphabets through SPSS software (version 21.0).

## 4. Conclusions

RSM was successfully used to optimize the different extraction variables of *P. stewartii* plant parts. The extraction yield increases with increasing extraction time, duration, and solvent concentration. HPLC analysis showed the presence of *p*-coumaric, gallic acid, quercetin, salicylic acid, sinapic acid, and vanillic acid as the main phytochemicals. The methanol extracts showed an overwhelming activity profile against α-glucosidase and α-amylase. It is concluded that extracts of *P. stewartii* could possess anti-inflammatory, immunomodulatory, antioxidant, hepatoprotective, and nephroprotective potential and serve as potential therapeutic agents in combating DM. Plans for this study must include in vivo trials of diseased models to explore the molecular mechanisms behind each bioactive component found in *P. stewartii* plant extracts.

## Figures and Tables

**Table 1:** Effect of independent variables on yield percentage.

Run	Solvent (mL)	Extraction Time (h)	Speed (rpm)	Yield (%)		
				LME	FME	WPME
1	200 (+1)	8 (+1)	150 (0)	8.97 ± 0.38 ^ab^	10.88 ± 0.28 ^d^	13.55 ± 0.11 ^fg^
2 (C_1_)	150 (0)	5 (0)	150 (0)	8.87 ± 0.25 ^d^	10.8 ± 0.21 ^e^	13.48± 0.18 ^c^
3 (C_2_)	150 (0)	5 (0)	150 (0)	8.86 ± 0.30 ^d^	10.79 ± 0.29 ^f^	13.48 ± 0.27 ^d^
4	150 (0)	8 (+1)	200 (+1)	8.96 ± 0.34 ^ab^	10.86 ± 0.19 ^ab^	13.54 ± 0.28 ^hi^
5	200 (+1)	5 (0)	200 (+1)	8.89 ± 0.34 ^c^	10.81 ± 0.22 ^fg^	13.49 ± 0.36 ^f^
6 (C_3_)	150 (0)	5 (0)	150 (0)	8.86 ± 0.24 ^df^	10.78 ± 0.31 ^c^	13.47 ± 0.41 ^m^
7 (C_4_)	150 (0)	5 (0)	150 (0)	8.87 ± 0.27 ^d^	10.78 ± 0.33 ^h^	13.47 ± 0.18 ^n^
8 (C_5_)	150 (0)	5 (0)	150 (0)	8.86 ± 0.31 ^d^	10.77 ± 0.23 ^i^	13.46 ± 0.30 ^e^
9	200 (+1)	2 (−1)	150 (0)	8.79 ± 0.19 ^fg^	10.73 ± 0.25 ^ba^	13.42 ± 0.34 ^a^
10	150 (0)	8 (+1)	100 (−1)	8.94 ± 0.28 ^b^	10.85 ± 0.18 ^a^	13.54 ± 0.16 ^hf^
11	150 (0)	2 (−1)	100 (−1)	8.77 ± 0.22 ^f^	10.71 ± 0.25 ^d^	13.41 ± 0.21 ^d^
12	100 (−1)	5 (0)	100 (−1)	8.84 ± 0.30 ^f^	10.76 ± 0.38 ^h^	13.44 ± 0.26 ^l^
13	100 (−1)	5 (0)	200 (+1)	8.85 ± 0.22 ^f^	10.77 ± 0.14 ^mn^	13.45 ± 0.31 ^q^
14	100 (−1)	8 (+1)	150 (0)	8.93 ± 0.33 ^bc^	10.83 ± 0.39 ^a^	13.52 ± 0.25 ^s^
15	100 (−1)	2 (−1)	150 (0)	8.76 ± 0.23 ^f^	10.71 ± 0.40 ^f^	13.41 ± 0.16 ^h^
16	200 (+1)	5 (0)	100 (−1)	8.88 ± 0.29 ^dk^	10.81 ± 0.29 ^k^	13.49 ± 0.32 ^d^
17	150 (0)	2 (−1)	200 (+1)	8.78 ± 0.12 ^fg^	10.72 ± 0.19 ^f^	13.42 ± 0.22 ^m^

C_1_–C_5_ = central points of yield extraction; Leaf methanol extract (LME); Flower methanol extract (FME); Whole plant methanol extract (WPME); ^a–q^ Means with different superscripts indicating the level of significant difference (*p* ≤ 0.05).

**Table 2:** ANOVA of the predicted second-order polynomial model through methanol mechanical shaking extraction conditions and effect on response parameters.

Source of Variation		Response Parameters						
		DF	Yield of LME		Yield of FME		Yield of WPME	
			MS	*p*-Value	MS	*p*-Value	MS	*p*-Value
Model		9	0.0072	0.0001	0.0046	0.0001	0.0036	0.0001
Linear Effects	A-Concentration	1	0.0028	0.0001	0.0005	0.0021	0.0017	0.0017
	B-Speed	1	0.0003	0.0060	0.2877	0.0001	0.4717	0.4717
	C-Time	1	0.0613	0.0001	0.0001	0.0300	0.0001	0.0001
Interaction Effects	AB	1	0.0000	1.0000	0.6044	0.0000	0.6074	0.6074
	AC	1	0.0000	0.3083	0.1478	0.0001	0.3178	0.3178
	BC	1	0.0000	0.3083	1.0000	0.0000	0.6074	0.6074
Quadratic Effects	A^2^	1	1.053 × 10^−6^	0.8281	0.5257	0.0001	0.4651	0.4651
	B^2^	1	1.053 × 10^−6^	0.8281	0.9145	4.211 × 10^−6^	0.8316	0.8316
	C^2^	1	0.0000	0.3972	0.9145	0.0002	0.1945	0.1945
Residual		7	0.0000	-	0.0001	-	0.0001	
Lack of Fit		3	8.333 × 10^−6^	0.8395	0.0000	0.8966	0.0001	0.3329
Pure Error		4	0.0000	-	0.0001		0.0001	
Cor. Total		16	-	-	-		-	

Leave methanol extract (LME), Flower methanol extract (FME), and Whole plant methanol extract (WPME).

**Table 3:** Regression equations of yield for actual and coded levels using RSM for methanol extraction.

Response Parameter	Regression Form	Regression Equation
Yield of LME	Coded	R1 = +8.86 + 0.0188A + 0.0063B + 0.0875C + 0.0000AB + 0.0025AC + 0.0025BC + 0.0005A^2^ + 0.0005B^2^ − 0.0020C^2^
	Actual	R1 = +8.67161 + 0.000232 Con − 0.000018 Speed +0.026389Time − 3.35785 × 10^−19^ Con * Speed + 0.000017 Con * Time + 0.000017 Speed * Time + 2.00000 × 10^−7^ Con^2^ + 2.00000 × 10^−7^ Speed^2^ − 0.000222 Time^2^
Yield of FME	Coded	R2 = +10.78 + 0.0200A + 0.0037B + 0.0687C − 0.0025AB + 0.0075AC + 0.0000BC + 0.0030A^2^ + 0.0005B^2^ + 0.0005C^2^
	Actual	R2 = +10.64606 − 0.000060Con + 0.000165Speed + 0.014861Time − 1.00000 × 10^−6^ Con * Speed + 0.000050 Con * Time + 2.78315 × 10^−20^ Speed * Time + 1.20000 × 10^−6^ Con^2^ + 2.00000 × 10^−7^ Speed^2^ + 0.000056 Time^2^
Yield of WPME	Coded	R3 = +13.47 + 0.0163A + 0.0025B + 0.0612C − 0.0025AB + 0.0050AC − 0.0025BC − 0.0035A^2^ − 0.0010B^2^ + 0.0065C^2^
	Actual	R3 = +13.28122 + 0.000728Con + 0.000403Speed + 0.010694Time − 1.00000 × 10^−6^ Con * Speed + 0.000033 Con * Time − 0.000017 Speed * Time − 1.40000 × 10^−6^ Con^2^ − 4.00000 × 10^−7^ Speed^2^ + 0.000722 Time^2^

A: Concentration; B: Speed; C: Time; AB: Concentration * Speed; AC: Concentration * Time; BC: Speed * Time; A^2^ Concentration^2^; B^2^ Speed^2^; C^2^ Time^2^; Leave methanol extract (LME); Flower methanol extract (FME); Whole plant methanol extract (WPME).

**Table 4:** Impact of mechanical shaking extraction conditions on the response parameters for TPC in methanol extracts.

Run	Solvent (mL)	Extraction Time (h)	Speed (rpm)	TPC (mg GAE/g DW)		
				LME	FME	WPME
1	200 (+1)	8 (+1)	150 (0)	19.51 ± 0.34 ^i^	13.15 ± 0.27 ^d^	17.39 ± 0.30 ^dk^
2 (C_1_)	150 (0)	5 (0)	150 (0)	19.11 ± 0.19 ^j^	12.88 ± 0.39 ^a^	17.12 ± 0.37 ^s^
3 (C_2_)	150 (0)	5 (0)	150 (0)	19.07 ± 0.32 ^kl^	12.87 ± 0.29 ^i^	17.13 ± 0.25 ^f^
4	150 (0)	8 (+1)	200 (+1)	19.48 ± 0.36 ^d^	13.11 ± 0.28 ^dj^	17.396 ± 0.38 ^i^
5	200 (+1)	5 (0)	200 (+1)	19.13 ± 0.29 ^ab^	12.89 ± 0.11 ^g^	17.19 ± 0.33 ^hi^
6 (C_3_)	150 (0)	5 (0)	150 (0)	19.01 ± 0.24 ^cj^	12.86 ± 0.37 ^a^	17.12 ± 0.13 ^i^
7 (C_4_)	150 (0)	5 (0)	150 (0)	18.99 ± 0.17 ^f^	12.91 ± 0.24 ^lj^	17.11 ± 0.27 ^gk^
8 (C_5_)	150 (0)	5 (0)	150 (0)	18.95 ± 0.39 ^f^	12.83 ± 0.18 ^c^	17.04 ± 0.20 ^w^
9	200 (+1)	2 (−1)	150 (0)	18.71 ± 0.21 ^h^	12.44 ± 0.11 ^e^	16.75 ± 0.21 ^k^
10	150 (0)	8 (+1)	100 (−1)	19.43 ± 0.30 ^hi^	13.14 ± 0.32 ^ij^	17.35 ± 0.17 ^ab^
11	150 (0)	2 (−1)	100 (−1)	18.68 ± 0.26 ^e^	12.43 ± 0.24 ^d^	16.71 ± 0.22 ^hi^
12	100 (−1)	5 (0)	100 (−1)	18.87 ± 0.19 ^df^	12.74 ± 0.22 ^g^	17.03 ± 0.31 ^ab^
13	100 (−1)	5 (0)	200 (+1)	18.91 ± 0.22 ^i^	12.81 ± 0.13 ^f^	17.02 ± 0.24 ^l^
14	100 (−1)	8 (+1)	150 (0)	19.41 ± 0.38 ^ij^	13.11 ± 0.33 ^hi^	17.33 ± 0.33 ^f^
15	100 (−1)	2 (−1)	150 (0)	18.64 ± 0.40 ^j^	12.39 ± 0.38 ^b^	16.64 ± 0.22 ^g^
16	200 (+1)	5 (0)	100 (−1)	19.16 ± 0.19 ^d^	12.88 ± 0.29 ^f^	17.16 ± 0.22 ^ik^
17	150 (0)	2 (−1)	200 (+1)	18.69 ± 0.24 ^m^	12.44 ± 0.25 ^d^	16.54 ± 0.24 ^y^

C_1_–C_5_ = central points of TPC; Total phenolic contents (TPC); Leaf methanol extract (LME); Flower methanol extract (FME); Whole plant methanol extract (WPME); ^a–y^ Means with different superscripts indicating the level of significant difference (*p* ≤ 0.05).

**Table 5:** ANOVA of the predicted second-order polynomial model through methanol mechanical shaking extraction conditions and influence on response parameter.

Source of Variation		Response Parameter						
		DF	TPC of LME		TPC of FME		TPC of WPME	
			MS	*p*-Value	MS	*p*-Value	MS	*p*-Value
Model		9	0.1421	0.0001	0.1143	0.0001	0.1195	0.0001
Linear Effects	A-Concentration	0.0578	0.0087	0.0120	0.0107	0.0276	0.0068	0.0068
	B-Speed	0.0006	0.7218	0.0004	0.5260	0.0014	0.4296	0.4296
	C-Time	1.21	0.0001	0.9870	0.0001	0.9983	0.0001	0.0001
Interaction Effects	AB	0.0012	0.6163	0.0009	0.3768	0.0004	0.6622	0.6622
	AC	0.0002	0.8286	0.0000	0.8795	0.0006	0.5865	0.5865
	BC	0.0004	0.7732	0.0004	0.5493	0.0117	0.0433	0.0433
Quadratic Effects	A^2^	0.0001	0.8705	0.0024	0.1692	0.0006	0.5845	0.5845
	B^2^	0.0000	0.9291	0.0011	0.3291	0.0011	0.4720	0.4720
	C^2^	0.0093	0.1918	0.0229	0.0021	0.0332	0.0043	0.0043
Residual		7	0.0045	-	0.0010	-	0.0019	-
Lack of Fit		3	0.0050	0.4123	0.0012	0.3558	0.0027	0.2506
Pure Error		4	0.0041	-	0.0008	-	0.0013	-
Cor. Total		16	-	-	-	-	-	-

Total phenolic contents (TPC); Leave methanol extract (LME); Flower methanol extract (FME); Whole plant methanol extract (WPME).

**Table 6:** Regression equations of TPC for actual and coded levels using RSM for methanolic extraction.

Response Parameter	Regression Form	Regression Equation
TPC of LME	Coded	R4 = +19.03 + 0.0850A + 0.0088B + 0.3888C0.0175AB + 0.0075AC + 0.0100BC − 0.0055A^2^ − 0.0030B^2^ + 0.0470B^2^
	Actual	R4 = +18.08089 + 0.003160Con + 0.001252Speed + 0.059861Time − 7.00000 × 10^−6^ Con * Speed + 0.000050 Con * Time + 0.000067 Speed * Time − 2.20000 × 10^−6^ Con^2^ − 1.20000 × 10^−6^ Speed^2^ + 0.005222Time^2^
TPC of FME	Coded	R5 = +12.87 + 0.0387A + 0.0075B + 0.3513C − 0.0150AB − 0.0025AC − 0.0100BC − 0.0237A^2^ − 0.0162B2 − 0.0738C^2^
	Actual	R5 = +11.38347 + 0.004608Con + 0.003333Speed + 0.211528Time − 6.00000 × 10^−6^ Con * Speed − 0.000017 Con * Time − 0.000067 Speed * Time − 9.50000 × 10^−6^ Con^2^ − 6.50000 × 10^−6^ Speed^2^ − 0.008194Tim^2^
TPC of WPME	Coded	R6 = +17.10 + 0.0588A − 0.0130B + 0.3533C + 0.0100AB0.0125AC + 0.0540BC + 0.0123A^2^ − 0.0162B^2^ − 0.0888C^2^
	Actual	R6 = +16.39297 − 0.000478Con − 0.000710Speed + 0.174861Time + 4.00000 × 10^−6^ Con * Speed − 0.000083 Con * Time + 0.000360 Speed * Time + 4.90000 × 10^−6^ Con^2^ − 6.50000 × 10^−6^ Speed^2^ − 0.009861Tim^2^

A: Concentration; B: Speed; C: Time; AB: Concentration * Speed; AC: Concentration* Time; BC: Speed * Time; A^2^ Concentration^2^; B^2^ Speed^2^; C^2^ Time^2^; Total phenolic contents (TPC); Leave methanol extract (LME); Flower methanol extract (FME); Whole plant methanol extract (WPME).

**Table 7:** Impact of mechanical shaking extraction conditions on the response parameters for TFC in methanol extracts.

Run	Solvent (mL)	Extraction Time (h)	Speed (rpm)	TFC (mg QE/g DW)		
				LME	FME	WPME
1	200 (+1)	8 (+1)	150 (0)	4.78 ± 0.34 ^a^	2.01 ± 0.33 ^p^	4.53 ± 0.12 ^g^
2 (C_1_)	150 (0)	5 (0)	150 (0)	4.61 ± 0.37 ^gh^	2.13 ± 0.24 ^f^	4.38 ± 0.23 ^ik^
3 (C_2_)	150 (0)	5 (0)	150 (0)	4.59 ± 0.24 ^b^	2.08 ± 0.10 ^l^	4.37 ± 0.20 ^de^
4	150 (0)	8 (+1)	200 (+1)	4.78 ± 0.28 ^c^	2.31 ± 0.40 ^t^	4.52 ± 0.25 ^df^
5	200 (+1)	5 (0)	200 (+1)	4.64 ± 0.14 ^ik^	2.16 ± 0.33 ^e^	4.42 ± 0.32 ^d^
6 (C_3_)	150 (0)	5 (0)	150 (0)	4.59 ± 0.18 ^e^	2.07 ± 0.38 ^m^	4.34 ± 0.17 ^c^
7 (C_4_)	150 (0)	5 (0)	150 (0)	4.58 ± 0.19 ^h^	2.07 ± 0.22 ^t^	4.32 ± 0.16 ^ba^
8 (C_5_)	150 (0)	5 (0)	150 (0)	4.57 ± 0.19 ^c^	2.03 ± 0.32 ^h^	4.29 ± 0.16 ^l^
9	200 (+1)	2 (−1)	150 (0)	4.42 ± 0.36 ^bc^	1.92 ± 0.21 ^f^	4.17 ± 0.20 ^s^
10	150 (0)	8 (+1)	100 (−1)	4.75 ± 0.28 ^c^	2.29 ± 0.38 ^i^	4.51 ± 0.31 ^kj^
11	150 (0)	2 (−1)	100 (−1)	4.37 ± 0.29 ^dc^	1.89 ± 0.15 ^a^	4.15 ± 0.38 ^b^
12	100 (−1)	5 (0)	100 (−1)	4.52 ± 0.17 ^di^	2.03 ± 0.21 ^k^	4.25 ± 0.32 ^d^
13	100 (−1)	5 (0)	200 (+1)	4.56 ± 0.18 ^i^	2.03 ± 0.23 ^hi^	4.26 ± 0.09 ^id^
14	100 (−1)	8 (+1)	150 (0)	4.75 ± 0.22 ^f^	2.26 ± 0.34 ^d^	4.47 ± 0.27 ^l^
15	100 (−1)	2 (−1)	150 (0)	4.34 ± 0.23 ^i^	1.85 ± 0.17 ^e^	4.11 ± 0.11 ^k^
16	200 (+1)	5 (0)	100 (−1)	4.62 ± 0.30 ^f^	2.14 ± 0.19 ^n^	4.41 ± 0.37 ^cd^
17	150 (0)	2 (−1)	200 (+1)	4.41 ± 0.10 ^i^	1.91 ± 0.24 ^ij^	4.16 ± 0.23 ^i^

C_1_–C_5_ = central points of TFC; Total flavonoid contents (TFC); Leaf methanol extract (LME); Flower methanol extract (FME); Whole plant methanol extract (WPME); ^a–s^ Means with different superscripts indicating the level of significant difference (*p* ≤ 0.05).

**Table 8:** ANOVA of the predicted second-order polynomial model through methanolic mechanical shaking extraction conditions influences response parameters for TFC.

Source of Variation		Response Parameter						
		DF	TFC of LME		TFC of FME		TFC of WPME	
			MS	*p*-Value	MS	*p*-Value	MS	*p*-Value
Model		9	0.0336	0.0001	0.0286	0.0203	0.0316	0.0003
Linear Effects	A-Concentration	1	0.0105	0.0005	0.7830	0.0242	0.0050	0.0050
	B-Speed	1	0.0021	0.0005	0.7830	0.0002	0.7245	0.7245
	C-Time	1	0.2888	0.2112	0.0004	0.2592	0.0001	0.0001
Interaction Effects	AB	1	0.0001	0.0001	0.8965	0.0000	1.0000	1.0000
	AC	1	0.0006	0.0256	0.0678	0.0000	1.0000	1.0000
	BC	1	0.0001	0.0000	1.0000	0.0000	1.0000	1.0000
Quadratic Effects	A^2^	1	0.0001	0.0061	0.3278	0.0004	0.6110	0.6110
	B^2^	1	0.004	0.0114	0.1932	0.0001	0.7978	0.7978
	C^2^	1	0.0006	0.0033	0.4637	0.0004	0.6110	0.6110
Residual		7	0.0002	-	0.0055	-	0.0015	-
Lack of Fit		3	0.0002	0.4712	0.0111	0.0317	0.0017	0.4070
Pure Error		4	0.0002	-	0..0013	-	0.0014	-
Cor. Total		16	-	-	-	-	-	-

Total phenolic contents (TPC); Leave methanol extract (LME); Flower methanol extract (FME); Whole plant methanol extract (WPME).

**Table 9:** Regression equations of TFC for actual and coded levels using RSM for methanolic extraction.

Response Parameter	Regression Form	Regression Equation
TFC of LME	Coded	R7 = +4.59 + 0.0363A + 0.0163B + 0.1900C − 0.0050AB − 0.0125AC − 0.0025BC − 0.0040A^2^ + 0.0010B^2^ − 0.0115C^2^
	Actual	R7 = +3.93489 + 0.001922Con + 0.000588Speed + 0.091111Time − 2.00000 × 10^−6^ Con * Speed − 0.000083 Con * Time − 0.000017 Speed * Time − 1.60000 × 10^−6^ Con^2^ + 4.00000 × 10^−7^ Speed^2^ − 0.001278Time^2^
TFC of FME	Coded	R8 = +2.08 + 0.0075A + 0.0075B + 0.1625C + 0.0050AB − 0.0800AC + 0.0000BC − 0.0380A^2^ + 0.0520B^2^ − 0.0280C^2^
	Actual	R8 = +1.45339Con − 0.006390Speed + 0.165278Time + 2.00000 × 10^−6^ Con * Speed − 0.000533Con * Time − 3.54202 × 10^−20^ Speed * Time − 0.000015Con^2^ + 0.000021Speed^2^ − 0.003111Time^2^
TFC of WPME	Coded	R9 = +4.34 + 0.0550A + 0.0050B + 0.1800C + 0.0000AB + 0.0000AC + 0.0000BC − 0.0100A^2^ + 0.0050B^2^ − 0.0100C^2^
	Actual	R9 = +3.78722 + 0.002300Con − 0.000500Speed + 0.071111time − 5.85380 × 10^−19^ Conc * Speed + 1.32185 × 10^−18^ Con * Time + 2.10381 × 10^−20^ Speed * Time − 4.00000 × 10^−6^ Con^2^ + 2.00000 × 10^−6^ Speed^2^ − 0.001111Time^2^

A: Concentration; B: Speed; C: Time; AB: Concentration * Speed; AC: Concentration * Time; BC: Speed * Time; A^2^ Concentration^2^; B^2^ Speed^2^; C^2^ Time^2^; Total flavonoid contents (TFC); Leave methanol extract (LME); Flower methanol extract (FME); Whole plant methanol extract (WPME).

**Table 10:** α-Amylase and α-glucosidase inhibitory effects of *P. stewartii* methanol extracts.

Sample	Concentration (µg/mL)	% of Inhibition α-Amylase	% of Inhibition α-Glucosidase	α-Amylase IC_50_ Value (µg/mL)	α-Glucosidase IC_50_ Value (µg/mL)
Acarbose	25	46.66 ± 0.30 ^d^	45.71 ± 0.29 ^d^	33.29 ± 0.34	37.29 ± 0.28
	50	55.01 ± 0.39 ^c^	54.22 ± 0.34 ^c^		
	100	66.22 ± 0.44 ^b^	65.12 ± 0.44 ^b^		
	200	88.11 ± 0.54 ^a^	87.18 ± 0.55 ^a^		
LME	25	44.27 ± 0.31 ^d^	43.08 ± 0.3 ^d^	46.86 ± 0.21	46.81 ± 0.17
	50	52.27 ± 0.34 ^c^	52.67 ± 0.35 ^c^		
	100	63.17 ± 0.45 ^b^	63.32 ± 0.47 ^b^		
	200	83.43 ± 0.58 ^a^	82.49 ± 0.57 ^a^		
WPME	25	41.72 ± 0.34 ^d^	41.91 ± 0.34 ^d^	53.88 ± 0.11	51.19 ± 0.30
	50	51.85 ± 0.34 ^c^	51.87 ± 0.34 ^c^		
	100	63.09 ± 0.34 ^b^	62.55 ± 0.34 ^b^		
	200	81.15 ± 0.54 ^a^	81.89 ± 0.54 ^a^		
FME	25	40.11 ± 0.34 ^d^	40.68 ± 0.34 ^d^	58.88 ± 0.12	56.68 ± 0.16
	50	50.77 ± 0.34 ^c^	50.77 ± 0.34 ^c^		
	100	62.08 ± 0.34 ^b^	61.28 ± 0.34 ^b^		
	200	81.88 ± 0.50 ^a^	80.22 ± 0.52 ^a^		

Leave methanol extracts (LME), Flower methanol extracts (FME), and Whole plant methanol extracts (WPME), ^a–d^ showing the level of significance (*p* ≤ 0.05).

**Table 11:** The actual and code levels of independent variables for optimized conditions (As estimated by BBD).

Independent Variable	Unit	Coded Level		
		−1	0	+1
Extraction time	Second	2	5	8
Speed	(m/s)	100	150	200
Solvent concentration	mL	100	150	200

## Data Availability

Data are contained within the article.

## Abbreviations

Response surface methodology (RSM), Box–Behnken design (BBD), High-performance liquid chromatography (HPLC), Total phenolic content (TPC), total flavonoids contents (TFC), medicinal plants (MPs), Leaves methanol extract (LME), Flower methanol extract (FME), Whole plant methanol extract (WPME), Galic acid (GA), superoxidase dismutase (SOD), catalase (CAT), glutathione (GSH), diabetes mellitus (DM), oxidative stress (OS), reactive oxidative species (ROS).
